# An exploratory clustering analysis of the 2016 National Financial Well-Being Survey

**DOI:** 10.1371/journal.pone.0309260

**Published:** 2024-09-06

**Authors:** Nathan Phelps, Adam Metzler

**Affiliations:** 1 Financial Wellness Lab, University of Western Ontario, London, Ontario, Canada; 2 Department of Mathematics, Wilfrid Laurier University, Waterloo, Ontario, Canada; UCL: University College London, UNITED KINGDOM OF GREAT BRITAIN AND NORTHERN IRELAND

## Abstract

This paper uses cluster analysis to explore the results of the 2016 National Financial Well-Being survey. Our analysis identifies four groups, two of which have very similar levels of financial well-being but markedly different objective financial situations. These findings indicate there is a systematic bias between financial well-being and objective financial situation. Although it is understood that these two constructs do not evaluate exactly the same thing, the difference in objective financial situation between the two groups suggests that, for large subsets of the American population, these constructs may be more different than the existing literature seems to suggest. This underscores the importance of considering both objective and subjective measures when assessing an individual’s overall financial situation.

## 1. Introduction

In 2016, the Consumer Financial Protection Bureau (CFPB) conducted its National Financial Well-Being Survey [1]. When describing the objectives of the survey, they stated that they wanted to provide the first documentation of the state of financial well-being (FWB) of Americans, both overall and in specific subpopulations, and that they wanted to use the data to test specific hypotheses about the ways financial knowledge (FK) and financial behavior (FB) relate to FWB. In alignment with their goals, the CFPB has now published reports describing the state of FWB in America [2] and testing hypotheses about relationships between various aspects of household finance [3]. The analyses conducted by the CFPB, however, did not include a potentially useful tool for reaching the goals they set to achieve with their data: clustering.

With respect to the CFPB’s first objective, specifically their desire to study subpopulations, clustering can be a very useful tool. Although studying subpopulations is common in household finance literature, clustering has not been widely adopted in the field. It has been used to explore financial survey data from North America (e.g., [4–6]) and Europe ([7, 8]), as well as investment trading data [9], but oftentimes subpopulations are based on demographics like age or race/ethnicity (e.g., [1, 10]). While there is value in studying those subpopulations, clustering can be used to identify subpopulations that transcend demographic lines, potentially unveiling insights not seen when grouping using demographics.

It is less clear how clustering can be used to address the CFPB’s second objective. Although clustering is not a good tool for formally testing hypotheses, it can be used to generate hypotheses (e.g., [11]) by revealing patterns that suggest the existence of relationships. If the hypotheses generated through a clustering analysis are inconsistent with results of another study, it may be worth investigating that study to see if its results could be incorrect. For example, in the CFPB’s analysis of the relationships between aspects of household finance [3], they created a conceptual model where FK and financial skill (FS) influence FB, FB influences both FWB and objective financial situation (OFS), and OFS influences FWB. They then performed a statistical analysis to test this model, ultimately deciding to remove FK because of its limited relationship with FB. If, however, the conceptual model is incorrect, this analysis could undervalue FK. A benefit of clustering is that the user does not need to specify such a model to perform their analysis.

Considering the massive impact finances have on the lives of many people (e.g., [12]) and the richness of the National Financial Well-Being Survey dataset, we felt it was worthwhile to explore this dataset using clustering to see if we could generate additional insights that have not been documented in the household finance literature. Here, we apply clustering to scores for FWB, FK, and FS, revealing four groups. The groups differ not only with respect to the features involved in the clustering, but also with respect to FB, OFS, and self-assessed knowledge (SAK). Two groups are nearly opposites of one another, with one very strong in all aspects of household finance that we considered and one very weak in all aspects. The other two groups generally lie more in the middle and are more nuanced. These two groups have very similar levels of FWB but markedly different OFS. In our view, the extent of the difference suggests that the gap between these constructs is larger than the existing literature suggests. In Section 2, we describe the data available to us through the 2016 National Financial Well-Being Survey; Section 3 outlines the clustering methodology we employed; Section 4 provides our results; Section 5 details a post-hoc analysis; Section 6 discusses the implications of our findings; and Section 7 concludes the paper.

## 2. Data

The data from the 2016 National Financial Well-Being Survey [1] consists of 6394 respondents in the USA, with over 100 questions asked to each participant. It covers a range of financial topics including questions about budgeting, savings, having money for basic necessities such as food or health care, ability to absorb a financial shock, knowledge about financial products, and ability to follow through on goals, as well as other related topics like numeracy, frugality, and self-control. Many of these questions involve responses on a Likert scale. Additionally, many demographic variables are provided such as age bands, household income bands, race/ethnicity, and education. Table 1 illustrates a breakdown of the population based on demographic groups. To allow their sample to represent the American population, the CFPB assigned each respondent a weight. These weights were created using a three-step process that involved age, race/ethnicity, gender, education, and household income, as well as other factors [1].

**Table 1:** Demographic characteristics of the survey sample.

Demographic group	Percentage in each demographic group
**Age**	
18–34	23.93
35–44	12.95
45–54	16.81
55–69	27.04
70+	19.27
**Race or ethnicity**	
White, non-Hispanic	70.35
Black, non-Hispanic	10.71
Other, non-Hispanic	5.25
Hispanic	13.68
**Gender**	
Male	52.42
Female	47.58
**Education**	
Less than high school	6.71
High school or GED	25.37
Some college or Associate’s degree	30.23
Bachelor’s degree	20.52
Graduate or professional degree	17.17
**Marital status**	
Married	59.84
Widowed	5.63
Divorced or separated	10.78
Never married	18.00
Living with partner	5.76
**Household income**	
Less than $30,000	19.16
$30,000-$49,999	16.91
$50,000-$74,999	18.08
$75,000-$99,999	14.94
$100,000+	30.92

The CFPB defined five components of household finance: FWB, FK, FS, OFS, and FB. They define FWB as having control over day-to-day, month-to-month expenses, having the capacity to absorb a financial shock, being on track to meet financial goals, and having the financial freedom to make the choices that allow enjoyment of life. FK refers to an ability to correctly answer objective questions related to topics such as investing, inflation, and insurance products. FS describes one’s ability to gain reliable information to facilitate financial decisions, process information to make sensible financial decisions, and execute financial decisions and adapt as necessary to ensure goal attainment. OFS is based on factors such as resources (e.g., savings, insurance), material hardships (e.g., running out of food, inability to afford health care), and credit standing. FB is composed of financial management (e.g., paying bills on time, staying within budget), planning (e.g., setting goals, consulting a budget), following through on financial intentions, and savings habits. The survey questions involved with measuring each of these constructs are available in S1 Table 1 in S1 File.

We note that the terminology used in household finance literature is not universally agreed upon; the precise interpretation of terms such as FWB varies across studies. We do not wish to take a stance on the “correct” interpretation of these terms but, to maintain consistency with the CFPB, we have adopted their definitions throughout this paper. As much as possible, we try to be consistent with respect to our uses of terminology in our study (e.g., FK always refers to an ability to answer objective questions). However, the inconsistency within household finance literature makes it challenging to be completely consistent. For example, when we refer to the findings of [12], the definition of FWB is not identical to the one we have adopted, but conceptually they are very similar. Also, we think it is noteworthy that the different components of household finance involve varying levels of subjectivity. Arguably, FK is the only completely objective component, as even OFS involves some subjectivity, such as through interpreting the meanings of “sometimes” and “often”. FWB, FS, and FB all involve substantial subjectivity.

For FWB [13], FK ([14, 15]), and FS [16], the publicly available dataset includes scores (two for FK) based on the individual questions. With the exception of one of the FK scores, these scores were developed using item response theory, so the weight of individual questions within each score is not equal. The FWB scale ranges from 14 to 95, the Lusardi and Mitchell FK scale ranges from 0 to 3, the Houts and Knoll FK scale ranges from -2.053 to 1.267, and the FS scale ranges from 3 to 89, although the range in the dataset is only 5 to 85. For all scales, a larger value (i.e., farther from zero if positive and closer to zero if negative) is better. The CFPB [3] developed scores for OFS and FB too but made only the responses to individual questions that compose the scores (i.e., not the scores themselves) publicly available.

## 3. Methodology

Clustering is an unsupervised machine learning approach whose goal is to place observations into meaningful groups, with similar observations belonging to the same group. In our case, the observations are survey respondents. We chose to cluster based on the scores for FWB, FK, and FS (recall that scores for OFS and FB were not available). There were two FK scales available, one with three questions [14] and the other with 10 questions [15]. We opted to use the 10-question scale because it was developed using item response theory and the additional questions should provide a more nuanced depiction of an individual’s FK. This scale’s marginal reliability, which can be interpreted in a similar fashion to Cronbach’s alpha, was 0.70 [15].

As an alternative to clustering on the scores, we could have clustered based on responses to individual survey questions like in some previous studies where scores were not directly available (e.g., [4, 5]). However, using the scores for clustering rather than the responses to individual questions offers multiple advantages. The scores were designed such that a score was still given to a person that neglected to answer one or more of the questions, allowing us to keep almost all the data without worrying about impacts of missing responses. Only nine of the 6394 observations were removed due to a missing score. In addition, the scores are not simply a sum of responses to each of the questions, so using the scores allows us to incorporate the relative importance of each question for each component. Scores also sometimes account for differences in age or administration method (e.g., [13]), while clustering based on the individual questions would ignore differences observed between these groups.

When clustering, there are several factors that must be considered. One must quantify the similarity (or dissimilarity) between observations by defining a distance measure and determining how far apart observations are from one another, choose a clustering algorithm, and select an approach for choosing the number of clusters. As described in *An Introduction to Statistical Learning* [17], a seminal data science and machine learning textbook, this is a very subjective process:

“In practice, we try several different choices, and look for the one with the most useful or interpretable solution. With these methods, there is no single right answer—any solution that exposes interesting aspects of the data should be considered.”

We considered both the K-means clustering algorithm [18] paired with Euclidean distance (after standardizing the scores) and the partitioning around medoids (PAM) clustering algorithm [19] paired with Gower’s distance [20]. We note that K-means is the traditional approach in our situation, as PAM is more commonly used when the data to be clustered has both numerical and categorical variables. (Recall that we are clustering on scores, which are numerical, as opposed to responses, which are a mix of ordinal and categorical.) We chose to consider PAM anyway based on the mindset that “any solution that exposes interesting aspects of the data should be considered” [17]. K-means was implemented using the kmeans function from the stats package (version 4.2.1) in R [21], with all default arguments used except for the number of initial configurations, which was set to 100. PAM was implemented using the pam function from the cluster package (version 2.1.4) using the default arguments.

To choose the number of clusters, we used a subjective process that involved considering multiple numbers of clusters (from two to 10) and looking to see which number(s) revealed the most insightful patterns in the data, as recommended by [17]. To guide us, we used a combination of prior research (e.g., [5]) and the output of NbClust [22], an R function that suggests a number of clusters based on several indices. These indices are often not in agreement with one another, reflecting the subjective nature of this problem.

Clustering identifies groups of similar respondents, but it does not tell us what differentiates the groups. Further analysis is required in order to determine how the clusters are different. To investigate the differences between clusters, we used principal components analysis (PCA) to reduce our dataset to two dimensions and created a colour-coded scatter plot. We also visually examined between-cluster variation in responses to individual questions. Studying responses to questions that were used to determine the scores we used in the clustering (i.e., “in-sample” questions) allows us to get a better sense of each cluster. However, studying questions whose responses had no direct influence on the clustering (i.e., “out-of-sample” questions) is perhaps more interesting. In particular, in our situation we have “out-of-sample” questions representing an individual’s FB and OFS. Since FB and OFS were not directly involved in the clustering, between-cluster variations in these aspects may indicate they have some relationship with FWB, FK, and FS. Depending on the nature of these patterns, these findings could be trivial (e.g., we might expect clusters with higher FWB to also have higher OFS), but they could also suggest the existence of less obvious relationships. To analyze the variation between clusters with respect to FB and OFS, we computed the mean occurrence frequency of several outcomes for people in each cluster. For some of our outcomes of interest (e.g., having life insurance, having a retirement account), the response is binary, so computing how often an outcome occurs is straightforward. For the Likert scale responses (e.g., setting financial goals, material hardships such as running out of food) with only a small number of possible values, we converted the numeric scales to binary variables to permit this way of thinking by defining a breakpoint in the scale for each question (e.g., ≥ 4 is encoded as 1, < 4 is encoded as 0). Details about the threshold for individual outcomes are available in S2 Table 1 in S2 File. In all cases, those who refused to answer a question were removed.

To determine the influence of demographics on cluster membership, we created bar plots for age, race/ethnicity, gender, education, marital status, household income, and presence of a financially dependent child. We created these plots in two different ways, one that shows the proportion of each of our groups that is composed of each demographic and one that shows the proportion of each demographic that falls into each of our groups.

We acknowledge that the clustering procedure we have described does not make use of the weights assigned to each respondent, which impacts the resulting clusters. As a robustness check, we implemented weighted K-means clustering, using hierarchical clustering to get the initial centroids. The hierarchical clustering was implemented using the hclust function with Ward’s method [23] in R. Likewise, our analyses of the data (e.g., when creating plots or computing mean occurrence frequencies) did not incorporate the weights. We have also analyzed the data with weights. In the event that the results were similar with and without weights, we have chosen to present our findings with the weights omitted for the sake of simplicity.

## 4. Results

**Fig 1:**
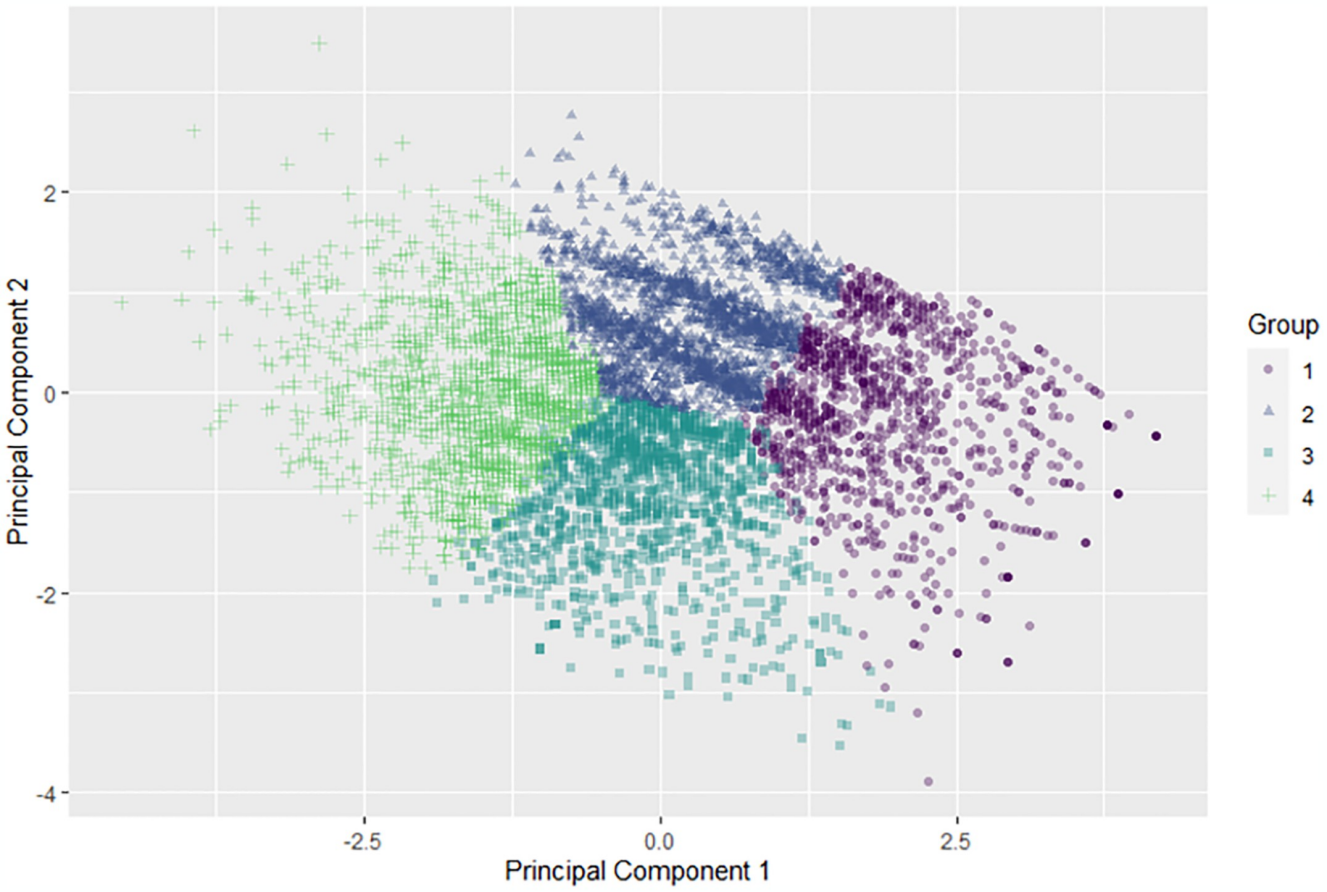
The four groups in principal component space.

The clustering results we found most intriguing came from asking the K-means algorithm to find four clusters. Summary statistics for these clusters are shown in Table 2. When incorporating the weights in the clustering, the resulting clusters (see S3 Fig 1 in S3 File) are nearly identical to the clusters obtained without weighting. Our analyses of the clusters also changed in only very minor ways by including the weights, so we have chosen to generally exclude the weights from the results we present. Thus, we are reporting unweighted statistics based on the survey sample, rather than weighted statistics based on the American population. However, S3 Table 1 in S3 File provides clustering summary statistics based on weights, so these can be used to make population-level statements. The similarities between our sample-level statistics and population-level statistics suggest the unweighted sample statistics can be treated as a rough estimate of the population-level statistics, but this should be done with caution.

**Table 2:** A summary of the four groups identified by clustering.

Group	Score			Size
	FWB	FS	FK	
1	74.4 (9.13)	65.0 (9.98)	0.53 (0.57)	1185 (19%)
2	56.5 (8.42)	48.7 (6.81)	0.56 (0.50)	2140 (34%)
3	56.2 (10.74)	57.4 (9.61)	-0.79 (0.43)	1305 (20%)
4	43.2 (9.95)	38.8 (7.87)	-0.65 (0.58)	1755 (27%)

The mean and standard deviation (in parentheses) of the scores for each group, as well as the size of each group. FWB, FS, and FK represent financial well-being, financial skill, and financial knowledge respectively. The scales’ possible values range from 14 to 95, 3 to 89, and -2.053 to 1.267 respectively. FWB and FS are based on answers to subjective questions, while FK is objective. All values in the table are based on calculations that do not consider the weights assigned to each respondent.

The results in Table 1 clearly show that Group 1 and Group 4 are completely different, as they fall on opposite ends for all three scores. Group 2 and Group 3 have almost the same mean FWB but differ with respect to their FS and FK. Based on their means, we have labeled the clusters in the following manner: 1) financially comfortable, skilled, and knowledgeable; 2) financially coping, somewhat skilled, and knowledgeable; 3) financially coping, fairly skilled, and unknowledgeable; and 4) financially stressed, unskilled, and unknowledgeable.

Fig 1 shows a scatter plot of the observations based on their first two principal components, which explain nearly 84% of the variation in the data. The associated eigenvectors are shown in S3 Table 2 in S3 File. The first principal component is computed using approximately equal weightings of all three features and completely separates Group 1 and Group 4. The second principal component separates Group 2 and Group 3 and can be loosely described as the difference between FK and FS.

**Fig 2:**
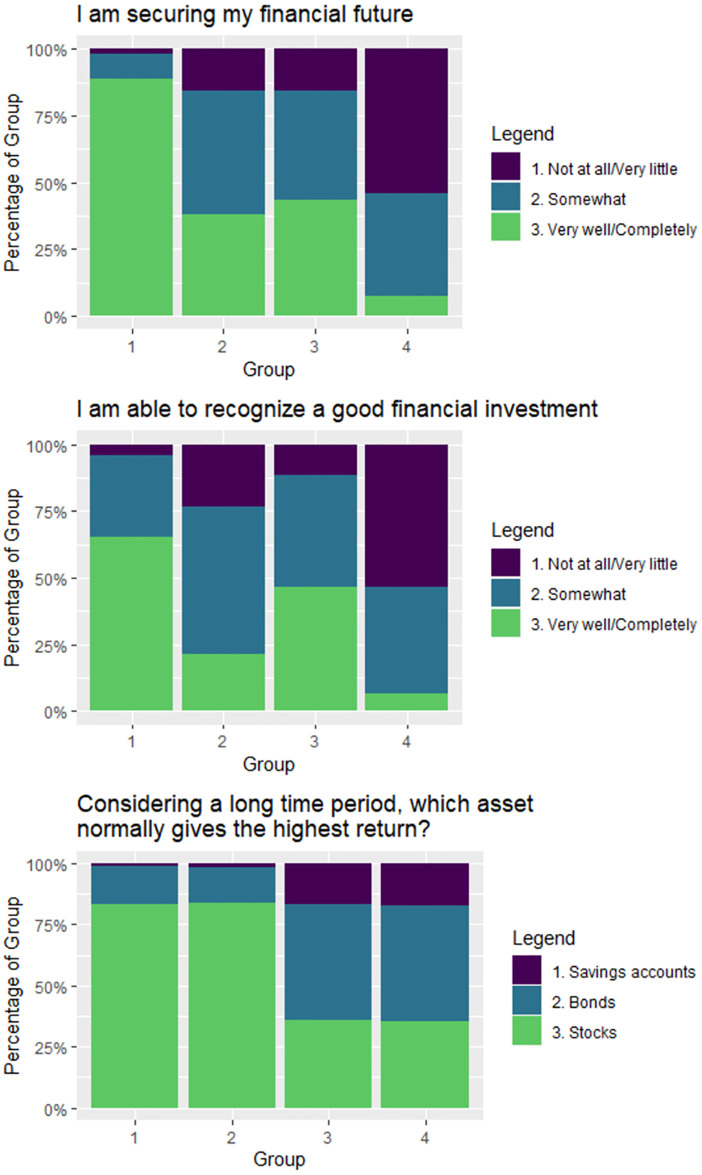
Responses to representative questions involved in clustering. These bar plots show responses by group to representative questions for financial well-being (top), financial skill (middle), and financial knowledge (bottom).

To give a clearer picture of what differentiates these groups, we have provided one representative question from each of the three scores involved in the clustering in Fig 2. As part of measuring FWB, respondents were asked how well the statement “I am securing my financial future” describes them. As expected, Group 1 feels most strongly that they are securing their future, while Group 4 is least confident. Group 2 and Group 3, who have similar FWB, answered this question similarly. Our representative question for FS asked respondents if the statement “I am able to recognize a good financial investment” describes them well. Unsurprisingly, Group 1 and Group 4 again responded most positively and most negatively respectively. Group 3’s confidence in recognizing a good financial investment is second only to Group 1’s, while Group 2’s confidence is third. This provides an interesting segue to our representative FK question, as the results from these two questions appear to contradict one another. Respondents were asked which of savings accounts, bonds, and stocks generally provide the best long-term returns. Group 1 and Group 2 responded very similarly, with nearly 85% correctly indicating stocks generally offer higher long-term returns. However, in both Group 3 and Group 4, only 35% answered this question correctly. For Group 3, responses to this question seem to contradict their self-reported ability to recognize a good financial investment. How can one recognize a good financial investment without understanding basic features of common investment options?

Table 3, which shows respondents’ SAK, sheds some light on this apparent contradiction. Respondents were asked to answer the question “How would you assess your overall financial knowledge?” Again, Group 1 and Group 4 are the two extremes, with Group 1 the most confident in their FK and Group 4 the least confident. However, despite Group 3 clearly having less actual FK (when measured objectively by the FK score), this group reports higher SAK than Group 2; over a quarter of them chose one of the two highest options, compared to about 16% of Group 3. This demonstrates a disconnect between what some people think they know and what they actually know, which helps explain the contradiction between responses to the FS and FK questions shown in Fig 2. Notably, Group 1 is also much more confident in their FK than Group 2, with over half of them choosing one of the two highest options even though the two groups have similar levels of FK.

**Table 3:** Self-assessment of overall financial knowledge by group.

Group	Self-assessment of overall financial knowledge						
	1	2	3	4	5	6	7
1	0.2	0.3	0.8	6.2	39.4	37.4	15.7
2	0.1	1.0	6.0	25.4	51.1	14.8	1.6
3	0.5	0.9	3.2	18.8	50.5	19.2	6.9
4	6.5	6.7	20.0	36.3	25.5	3.9	1.2

The percentage (rounded to one decimal place) of each group that provided the indicated self-assessment of their overall financial knowledge, excluding those who refused to respond. The scale goes from 1 (very low) to 7 (very high).

In Table 4, we show the percentage within each group that experienced several outcomes related to FB and OFS. It is evident that Group 1 and Group 4 are generally the “bookend” groups again. Broadly speaking, Group 1 has the best FB and OFS, although they report consulting their budget and actively considering steps to stick to their budget less than Group 3. The table illustrates the extent to which Group 4 is struggling. For example, only 15% of them followed through on financial goals they set for themselves and a startling 41% reported “sometimes” or “often” running out of food and not having money for more. With respect to FB, Group 2 and Group 3 are similar in some ways, but Group 3 appears to be much more involved in planning (e.g., budgeting) and followed through on financial goals they set for themselves more than Group 2. With respect to OFS, Group 2 experienced better outcomes across the board than Group 3. Given the similar FWB of these two groups, this is somewhat surprising, especially because the difference between the groups is dramatic for some outcomes. For example, the percentages of people that ran out of food and that were unable to afford housing are both three times larger for Group 3 than Group 2. Also, relative to Group 3, an additional 20%+ of Group 2 have a retirement account, can raise $2000 in 30 days, and have liquid savings of $5000 or more.

**Table 4:** Financial behavior and objective financial situation by group.

Construct	Response	Percentage experiencing outcome			
		Group 1	Group 2	Group 3	Group 4
Financial behavior	I consult my budget	64.2	63.3	79.0	55.9
	I actively consider steps to stick to budget	70.8	61.9	77.0	45.7
	I set financial goals	82.1	65.6	76.6	41.5
	I prepare a plan to achieve financial goals	61.6	37.8	59.9	22.5
	I follow through on financial commitments to others	98.7	91.2	88.1	56.4
	I follow through on financial goals I set for myself	97.1	56.3	74.9	15.0
	I paid all my bills on time	99.7	95.2	90.9	68.6
	I stayed within budget	91.2	72.8	80.7	35.3
	I paid off my credit card balance each month	91.2	66.0	59.5	23.4
	I checked my statements, etc. for errors	92.7	81.0	86.9	60.7
	Putting money into savings is a habit	95.0	80.6	85.1	48.8
Objective financial situation	Worried about running out of food*	0.0	6.1	17.8	44.6
	Ran out of food*	0.3	4.4	15.2	40.7
	Could not afford housing*	0.1	2.9	9.7	22.3
	Could not afford health care*	0.8	9.3	15.5	35.8
	Could not afford medication*	2.0	8.8	12.7	31.8
	Utilities shutdown*	0.2	1.4	7.2	17.8
	Has life insurance	63.7	60.7	44.8	38.2
	Has health insurance	85.0	82.3	62.5	55.4
	Has retirement account	83.1	72.7	44.5	32.1
	Has non-retirement investments	61.8	41.1	18.9	8.8
	Can raise $2000 in 30 days	97.6	76.1	55.1	20.8
	Rejected when applying for credit*	1.4	5.0	10.8	21.8
	Did not apply for credit because of anticipated rejection*	0.8	6.2	10.7	28.6
	Contacted by debt collector*	2.6	9.7	15.2	36.9
	Can make ends meet	98.3	70.8	65.5	21.7
	Has liquid savings of $5000 or more	90.6	66.0	45.0	16.4

The percentage of each group that experienced various financial outcomes related to financial behavior and objective financial situation, rounded to one decimal place. Negative outcomes are denoted with an asterisk.

Two other observations also stood out to us. With the exception of those in Group 1, many Americans are not paying their credit card in full each month. Even for Group 2 (who are financially knowledgeable and have a relatively strong OFS), only 66% of people reported paying their credit card in full, and for Group 4 it drops to 23%. Given the exorbitant interest rates on credit card debt, it is noteworthy how many people carry this type of debt. Secondly, the four groups differ dramatically in their rates of having health insurance, even though nobody is impervious to medical issues. Group 1 is the most insured (85%), Group 2 is second (82%), followed by Group 3 (63%), and finally Group 4 (55%). We recognize that insurance is not affordable for some people, which might be a cause of the different levels of insurance within the groups. However, those unable to afford insurance are also those who need it most since they do not have the financial resources to fall back on in an emergency.

Bar plots investigating the demographics of our groups are shown in S3 Figs 2–8 in S3 File, showing that members of certain demographic groups are over- and under-represented in certain clusters. For example, 38% of those aged 18–24 are in Group 4, compared to 6.5% in Group 1; 40% of Black and Hispanic people are in Group 4, compared to 9.8% in Group 1; and 54% of those with a household income below $20,000 are in Group 4, compared to 2.5% in Group 1. However, there are people from every demographic group in each cluster, so demographics are clearly not the sole determinant of cluster membership. Thus, these clusters could not have been found using the more traditional approach of splitting the data by factors such as age, gender, race/ethnicity, or household income (e.g., [1, 10]).

## 5. Post-hoc analysis

One of the clear differences across our groups is the varying levels of FK and SAK, which motivates a closer look at the gap between what people think they know and what they actually know. To quantify the gap between SAK and actual FK, we computed the quantile for each respondent’s SAK and actual FK and subtracted the former from the latter, creating a score that ranges from negative one (very overconfident) to one (very underconfident). For example, if an individual’s SAK is greater than or equal to 65% of the population, while their FK score is greater than or equal to 30% of the population, then their score is -0.35. The computation included respondents that refused to provide a self-assessment of their knowledge (less than 1% of survey respondents), whose responses were encoded as negative one. For Group 1 and Group 4, the average gap is -0.05 and -0.07 respectively, indicating that the respondents in these groups provide reasonable self-assessments of their knowledge on average. Group 2’s average gap is 0.16, indicating an underestimation of their FK, while Group 3 (which has more FS than Group 2 but less FK) dramatically overestimates their FK, as shown by their average gap of -0.42. The gap between SAK and actual FK could be partially caused by demographic differences; when people provide their SAK, they think about what they know compared to what others around them know. Compared to Group 2, Group 3 is less educated, earns less income, and is composed of more ethnic/racial minorities and women. Regardless of its cause, this overestimation of FK can be dangerous, especially as people age and become more vulnerable to financial abuse [24].

## 6. Discussion

Using K-means clustering on an American dataset with scores for FWB, FK, and FS, we have identified four clusters. Group 1 and Group 4 consistently fall on opposite ends of the spectrum, with Group 1 on the better end (i.e., better FWB, more FK, etc.) and Group 4 on the worse end. This is even true for OFS, FB, and SAK, which are not involved in the clustering procedure. Although it is well-known that many Americans struggle financially, the financial situation of those in Group 4 is a reminder of the severity of this struggle, with a substantial percentage of them struggling to afford food, housing, and/or health care. Group 2 and Group 3 are faring significantly better than Group 4, but not to the same degree; although they have very similar levels of FWB, Group 2 has a considerably better OFS than Group 3.

It is well-recognized in the literature that FWB and OFS are not identical constructs. However, it does not appear that the magnitude of the difference between such constructs is well understood. For example, the CFPB analyzed the relationship between FWB and OFS, finding that OFS explained 67% of the variance in FWB and concluding that the two constructs are “closely aligned” [3]. Based on the high R^2^ and strong language, readers could be forgiven for concluding that FWB and OFS are reasonable substitutes for one another. However, the similar levels of FWB for Group 2 and Group 3 combined with the substantial gap in OFS between the two suggests that this is not the case.

OFS represents a more robust measurement of the financial resilience of an individual since it is less subjective than FWB. In support of this view, based on a definition used in previous studies (e.g., [25, 26]), about three times as many people are “financially fragile” in Group 3 than in Group 2 (Those who said they could “probably not” or “certainly not” come up with $2000 if an unexpected need arose within the next month are defined as “financially fragile”. Ignoring those who refused to respond and those who said “I don’t know”, 7.4% of Group 2 and 20.9% of Group 3 are considered financially fragile.). Researchers and policymakers must carefully ensure that what they are measuring corresponds to what they are interested in studying. If they are truly interested in OFS but use FWB to measure it (or vice-versa), they run the risk of introducing a substantial amount of unnecessary error. In our view, if a policymaker is interested in making sure the population is financially resilient, they should not focus on FWB at the expense of OFS. Similarly, if a policymaker is interested in mental health or overall well-being, they should not focus on OFS at the expense of FWB.

While using FWB to measure OFS (and vice-versa) clearly introduces unnecessary error, there is still a question of the nature of this error. Is it solely random noise or does using one to measure the other introduce a systematic error (i.e., bias)? If it’s only noise, then the error will “wash out” in large samples, but a large sample does nothing to help mitigate a systematic error. In [27], the authors found that FK, internal locus of control, and psychological resilience have different relationships with objective and subjective financial fragility. This suggests that there may be a systematic error between objective and subjective constructs. However, when the CFPB modelled FWB as a function of OFS, they included several potential confounders (i.e., financial self-efficacy, frugality, perceived economic mobility, self-control, income, and retirement status) and found that none helped further explain much of the variability in FWB [3], which might suggest that the error is mostly random noise. In our case, it is important to note that OFS was not involved in our clustering procedure. The similar FWB yet different FK, FS, and OFS observed between Group 2 and Group 3 suggests that the relationship between FWB and OFS is influenced by FK and/or FS, or some other construct that is related to FK and/or FS (e.g., SAK, which has a Pearson correlation of 0.63 with FS). Thus, our results are consistent with [27]. This influence, whether it comes from FK, FS, or some other confounding factor, leads to a systematic bias in FWB as a measure of OFS (and vice-versa). If a researcher or policymaker uses FWB to measure OFS (and vice-versa), they not only introduce unnecessary error to their analysis, but also run the risk of biasing their analysis.

The systematic bias between OFS and FWB underscores the importance of considering both objective and subjective constructs when assessing an individual’s overall situation (e.g., [28]). This is also evident from the difference between FK and SAK; the Pearson correlation between the two is only 0.23, and we have seen that the gap between them can be substantial (see Section 5). Given the gaps we’ve observed between objective and subjective constructs that measure similar things, it is natural to question the reliability of responses to questions on topics like FB, which are very subjective. It may be the case that Group 3’s FBs are not better than Group 2’s in reality—they just think that they are. Investigating this through collecting more objective data on FB may be worthwhile.

Our clustering results also provide motivation for future studies focused on other relationships between FK, FS, FB, SAK, FWB, and OFS. As mentioned, the fact that the clusters differ with respect to constructs that were not involved in the clustering suggests the existence of relationships between these constructs. Most notably, the two groups with the best OFS are also the two most knowledgeable groups. This suggests that the CFPB’s conclusion that FK should be removed from their conceptual model [3] may be worth revisiting. The clustering analysis is far from definitive evidence of a relationship between FK and OFS because it does not control for confounding variables, but it does provide justification for conducting another regression analysis—perhaps without some of the assumptions imposed by the CFPB.

At the surface, some of our findings may appear redundant given the results presented in [27]. In particular, [27] showed that "high objective financial fragility does not necessarily overlap with high subjective financial fragility”, that there is a bias between these two constructs based on both demographics and cognitive and non-cognitive factors, and that FK helps mitigate objective financial fragility. However, there are multiple differences between the studies. First, the data from the studies were obtained over different time periods and from different continents. More importantly, the measurement of objective financial fragility in [27] is based on only a single question, rather than the much more exhaustive list of questions the CFPB used to measure OFS. By considering many more components of OFS, we have been able to improve our understanding of the bias between subjective and objective measures of an individual’s finances. In addition, when using this presumably more robust score for OFS, the CFPB concluded that FWB and OFS are “strongly linked” and that FK has very little relation to FB (and, by extension, OFS) [3]. To our knowledge, when using this score, nobody has illustrated how much variation in OFS can exist for a large subset of the American population with similar FWB, nor has anyone provided cause to question if there may in fact be some relationship between FK and OFS in this dataset (although further analysis is needed to see if this is the case).

Although our clustering analysis has revealed interesting insights, we note that the clusters are not separated by low-density zones (see Fig 1). If one wishes to use clustering to identify groups that will respond well to differing interventions or policies, the lack of low-density zones may pose a problem because people that fall near the boundaries might not be receptive to interventions designed for their assigned group. An alternative clustering approach that explicitly identifies people near the cluster boundaries (and thus facilitates targeting these individuals with unique interventions) such as fuzzy c-means might be more suitable for targeted interventions.

## 7. Conclusion

Using data from a 2016 American survey, we have used clustering to find four groups of people with very different characteristics. We have labeled these groups as 1) financially comfortable, skilled, and knowledgeable; 2) financially coping, somewhat skilled, and knowledgeable; 3) financially coping, fairly skilled, and unknowledgeable; and 4) financially stressed, unskilled, and unknowledgeable. These groups differ not only with respect to the aspects we clustered on, namely FWB, FS, and FK, but also with respect to other aspects, namely OFS, FB, and SAK. Group 4 demonstrates the challenging situation faced by many Americans, with a substantial proportion of them struggling to afford food or housing. Group 2 and Group 3 illustrate a substantial disconnect between FWB and OFS because they have a similar level of FWB but a very different OFS. In our opinion, if interested in studying how someone feels about their finances, then it seems FWB is most appropriate. If interested in studying someone’s financial resilience, we believe OFS is most appropriate. Choosing the “wrong” one can lead to inaccurately characterizing the situation of many individuals because of the systematic bias between FWB and OFS. The ways in which the four groups differ with respect to several components of household finance, including some not involved in the clustering, suggests there may be relationships between these components. In particular, the two groups with the best OFS also have the highest levels of FK. Notably, the suggestion of a meaningful relationship between these two constructs counters the findings of [3]. Further study of this dataset using regression analyses may affirm or oppose these findings, as well as provide insights into other relationships.

## Supporting information

S1 File Survey questions. Survey questions used to measure constructs of household finance.
(DOCX)

S2 File Converting Likert scale responses to binary responses. Describing how we converted responses to Likert scale survey questions to binary responses so they could be succinctly summarized.
(DOCX)

S3 File Additional clustering results. Weighted clustering results, principal components analysis, and demographic information for the clusters.
(DOCX)
