# Supplementary material for: An exploratory clustering analysis of the 2016 National Financial Well-Being Survey
Source: PLoS One. 2024 Sep 6;19(9):e0309260. doi: 10.1371/journal.pone.0309260 (PMC11379153; doi:10.1371/journal.pone.0309260)
Supplement: S1 File — Survey questions used to measure constructs of household finance. (DOCX) [file pone.0309260.s001.docx]

**S1 Table 1. Survey questions.**

| Construct | Question |
| --- | --- |
| Financial well-being | I could handle a major unexpected expense. |
|  | I am securing my financial future. |
|  | Because of my money situation, I feel like I will never have the things I want in life. |
|  | I can enjoy life because of the way I’m managing my money. |
|  | I am just getting by financially. |
|  | I am concerned that the money I have or will save won’t last. |
|  | Giving a gift for a wedding, birthday or other occasion would put a strain on my finances for the month. |
|  | I have money left over at the end of the month. |
|  | I am behind with my finances. |
|  | My finances control my life. |
| Financial knowledge (Houts and Knoll’s scale) | Imagine that the interest rate on your savings account was 1% per year and inflation was 2% per year. After 1 year, how much would you be able to buy with the money in this account? |
|  | Considering a long time period (e.g., 10-20 years), which asset normally gives the highest return: savings accounts, bonds, or stocks? |
|  | Normally, which asset displays the highest fluctuations over time: savings accounts, bonds, or stocks? |
|  | When an investor spreads their money among different assets, does the risk of losing a lot of money increase, decrease, or stay the same? |
|  | If you were to invest $1000 in a stock mutual fund, it would be possible to have less than $1000 when you withdraw your money. |
|  | Whole life insurance has a savings feature while term does not. |
|  | Housing prices in the US can never go down. |
|  | Suppose you owe $3000 on your credit card. You pay a minimum payment of $30 each month. At an Annual Percentage Rate of 12% (or 1% per month), how many years would it take to eliminate your credit card debt if you made no additional new charges? |
|  | If interest rates rise, what will typically happen to bond prices? |
|  | A 15-year mortgage typically requires higher monthly payments than a 30-year mortgage, but the total interest paid over the life of the loan will be less. |
| Financial knowledge (Lusardi and Mitchell’s scale) | Suppose you had $100 in a savings account and the interest rate was 2% per year. After 5 years, how much do you think you would have in the account if you left the money to grow? |
|  | Imagine that the interest rate on your savings account was 1% per year and inflation was 2% per year. After 1 year, how much would you be able to buy with the money in this account? |
|  | Buying a single company’s stock usually provides a safter return than a stock mutual fund. |
| Financial skill | I know how to get myself to follow through on my financial intentions. |
|  | I know where to find the advice I need to make decisions involving money. |
|  | I know how to make complex financial decisions. |
|  | I am able to make good financial decisions that are new to me. |
|  | I am able to recognize a good financial investment. |
|  | I know how to keep myself from spending too much. |
|  | I know how to make myself save. |
|  | I know when I do not have enough information to make a good decision involving money. |
|  | I know when I need advice about my money. |
|  | I struggle to understand financial information. |
| Financial behavior | I consult my budget to see how much money I have left. |
|  | I actively consider the steps I need to take to stick to my budget. |
|  | I set financial goals for what I want to achieve with my money. |
|  | I prepare a clear plan of action with detailed steps to achieve my financial goals. |
|  | I follow through on my financial commitments to others. |
|  | I follow through on financial goals I set for myself. |
|  | I paid all my bills on time. |
|  | I stayed within my budget or spending plan. |
|  | I paid off my credit card balance in full each month. |
|  | I checked my statements, bills, and receipts to make sure there were no errors. |
|  | Putting money into savings is a habit for me. |
| Objective financial situation | I worried whether our food would run out before I got money to buy more. |
|  | The food that I bought just didn’t last and I didn’t have money to get more |
|  | I couldn’t afford a place to live. |
|  | I or someone in my household needed to see a doctor or go to the hospital but didn’t because we couldn’t afford it. |
|  | I or someone in my household stopped taking medication or took less than directed due to costs. |
|  | One or more of my utilities was shut off due to non-payment. |
|  | How confident are you that you could come up with $2000 in 30 days if an unexpected need arose? |
|  | How much do you have in savings today (in cash, checking, and savings account balances)? |
|  | How many financial products do you own (e.g., checking/savings account, life insurance, pension, etc.)? |
|  | Were you contacted in the past year by a person or company trying to collect past-due debt? |
|  | Were you turned down for credit in the past year? |
|  | Did you choose not to apply for credit in the past year because you thought you’d get turned down? |
|  | In a typical month, how difficult is it for you to cover your expenses and pay all your bills? |

Survey questions asked about each of the five constructs of household finance defined by the Consumer Financial Protection Bureau (CFPB). Some of the questions have been paraphrased.
