# Supplementary material for: An exploratory clustering analysis of the 2016 National Financial Well-Being Survey
Source: PLoS One. 2024 Sep 6;19(9):e0309260. doi: 10.1371/journal.pone.0309260 (PMC11379153; doi:10.1371/journal.pone.0309260)
Supplement: S2 File — Describing how we converted responses to Likert scale survey questions to binary responses so they could be succinctly summarized. (DOCX) [file pone.0309260.s002.docx]

**S2 Table 1. Initial scale and binary encoding of survey questions.**

| Survey question | Initial scale and binary encoding |
| --- | --- |
| I consult my budget to see how much money I have left.  I actively consider the steps I need to take to stick to my budget.  I set financial goals for what I want to achieve with my money.  I prepare a clear plan of action with detailed steps to achieve my financial goals.  Putting money into savings is a habit for me. | These were measured on a scale from 1 (strongly disagree) to 5 (strongly agree). Responses of 4 or 5 were considered a “yes” (encoded as 1) and remaining responses were considered a “no” (encoded as 0). |
| I follow through on my financial commitments to others.  I follow through on financial goals I set for myself. | These were measured on a scale from 1 (not at all) to 5 (very well). Responses of 4 or 5 were considered a “yes” (encoded as 1) and remaining responses were considered a “no” (encoded as 0). |
| I paid all my bills on time.  I stayed within my budget or spending plan.  I paid off my credit card balance in full each month.  I checked my statements, bills, and receipts to make sure there were no errors. | These were measured on a scale from 1 (not applicable or never) to 5 (always). Responses of 4 or 5 were considered a “yes” (encoded as 1) and remaining responses were considered a “no” (encoded as 0). |
| I worried whether our food would run out before I got money to buy more. | These were measured on a scale from 1 (never) to 3 (often). Responses of 1 were considered a “no” (encoded as 0) and remaining responses were considered a “yes” (encoded as 1). |
| The food that I bought didn’t last and I didn’t have money to get more. |  |
| I couldn’t afford a place to live. |  |
| I or someone in my household needed to see a doctor or go to the hospital but did not go because we couldn’t afford it. |  |
| I or someone in my household stopped taking a medication or took less than directed due to the costs. |  |
| One or more of my utilities was shut off due to non-payment. |  |
| How confident are you that you could come up with $2000 in 30 days if an unexpected need arose within the next month? | This was measured on a scale from 1 (certainly could not) to 4 (certainly could) and included an “I don’t know” option. Responses of 4 were considered a “yes” (encoded as 1) and remaining responses were considered a “no” (encoded as 0). |
| In the past 12 months, have you been contacted by a person or company trying to collect past-due debt from you? | Respondents had the options of “yes”, “no”, and “not sure”. Responses of “yes” and “not sure” were encoded as 1, while “no” was encoded as 0. |
| In a typical month, how difficult is it for you to cover your expenses and pay all your bills? | This was measured on a scale from 1 (not at all difficult) to 3 (very difficult). Responses of 1 were considered an ability to easily make ends meet and encoded as 1, while other responses were encoded as 0. |
| How much money do you have in savings today (in cash, checking, and saving account balances)? | Respondents were provided with several ranges as options. We encoded those that reported savings of $5000 or more as 1 and others as 0. Those that didn’t know or preferred not to say were removed. |

This table details (paraphrased) survey questions, their initial scale, and how they were encoded as a binary variable. For all questions, those who refused to answer the question were removed.
