# Supplementary material for: An exploratory clustering analysis of the 2016 National Financial Well-Being Survey
Source: PLoS One. 2024 Sep 6;19(9):e0309260. doi: 10.1371/journal.pone.0309260 (PMC11379153; doi:10.1371/journal.pone.0309260)
Supplement: S3 File — Weighted clustering results, principal components analysis, and demographic information for the clusters. (DOCX) [file pone.0309260.s003.docx]

**S3 Table 1. Summary statistics.**

| Group | Weighted Mean | | | Weighted Size |
| --- | --- | --- | --- | --- |
|  | FWB | FS | FK |  |
| 1 | 73.4 | 65.5 | 0.483 | 953 (15%) |
| 2 | 55.9 | 48.4 | 0.523 | 1922 (30%) |
| 3 | 55.6 | 57.6 | -0.829 | 1454 (23%) |
| 4 | 42.9 | 38.7 | -0.678 | 2057 (32%) |

The weighted mean of the scores for each group and weighted size of each group, based on survey weights defined by the Consumer Financial Protection Bureau (CFPB). FWB, FS, and FK represent financial well-being, financial skill, and financial knowledge respectively. The scales’ possible values range from 14 to 95, 3 to 89, and -2.053 to 1.267 respectively. FWB and FS are based on answers to subjective questions, while FK is objective. All values in the table are based on calculations that do not consider the weights assigned to each respondent.

**S3 Table 2. Eigenvectors for each score on each principal component.**

| Score | Principal Component 1 | Principal Component 2 | Principal Component 3 |
| --- | --- | --- | --- |
| Financial well-being | 0.651 | -0.146 | 0.745 |
| Financial skill | 0.595 | -0.513 | -0.619 |
| Financial knowledge | 0.472 | 0.846 | -0.247 |

**S3 Fig 1. Robustness check with weighted clustering.**


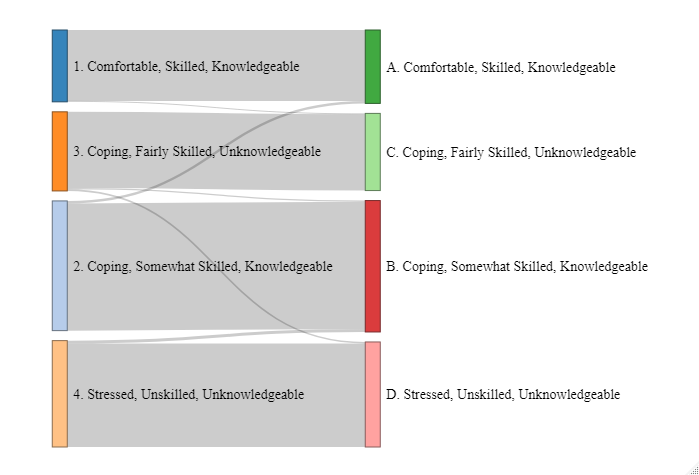


This is a Sankey diagram showing the movement of respondents from their group in the clustering results presented (left) to their group based on weighted clustering (right).

**S3 Fig 2. Associations between age and group.**


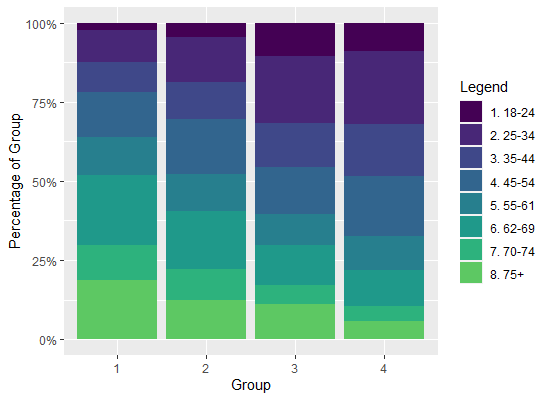

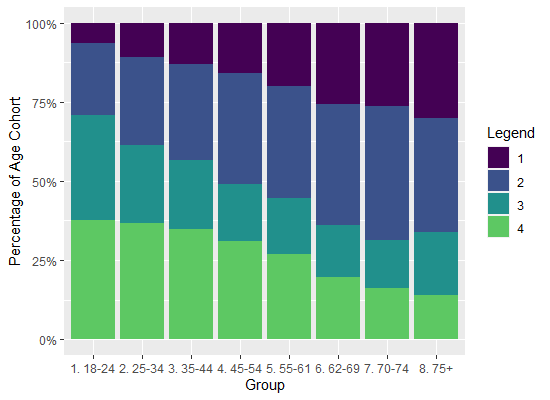


The left bar plot shows the percentage of each group belonging to each age cohort and the right plot shows the percentage of each age cohort belonging to each group.

**S3 Fig 3. Associations between race/ethnicity and group.**


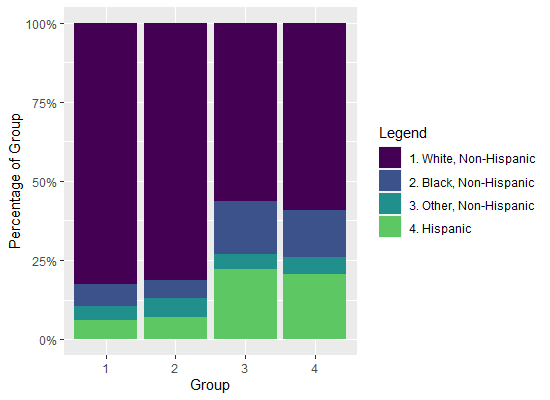

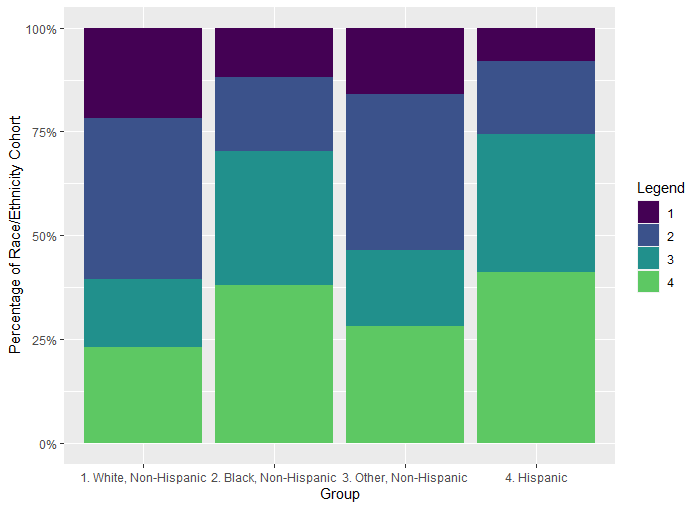


The left bar plot shows the percentage of each group belonging to each race/ethnicity cohort and the right plot shows the percentage of each race/ethnicity cohort belonging to each group.

**S3 Fig 4. Associations between gender and group.**


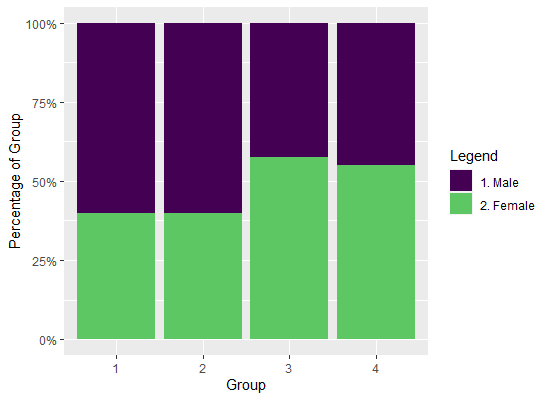

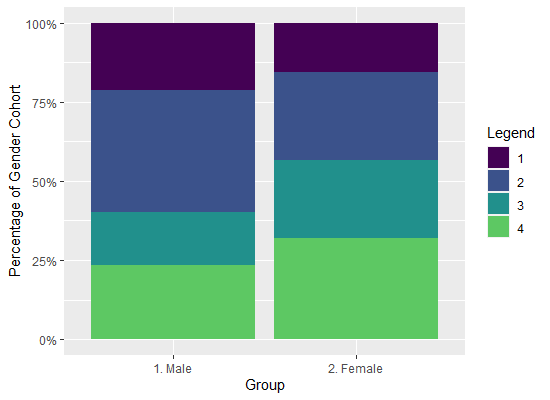


The left bar plot shows the percentage of each group belonging to each gender cohort and the right plot shows the percentage of each gender cohort belonging to each group.

**S3 Fig 5. Associations between education and group.**


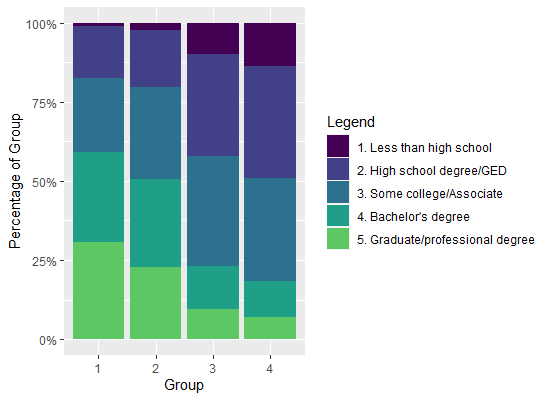

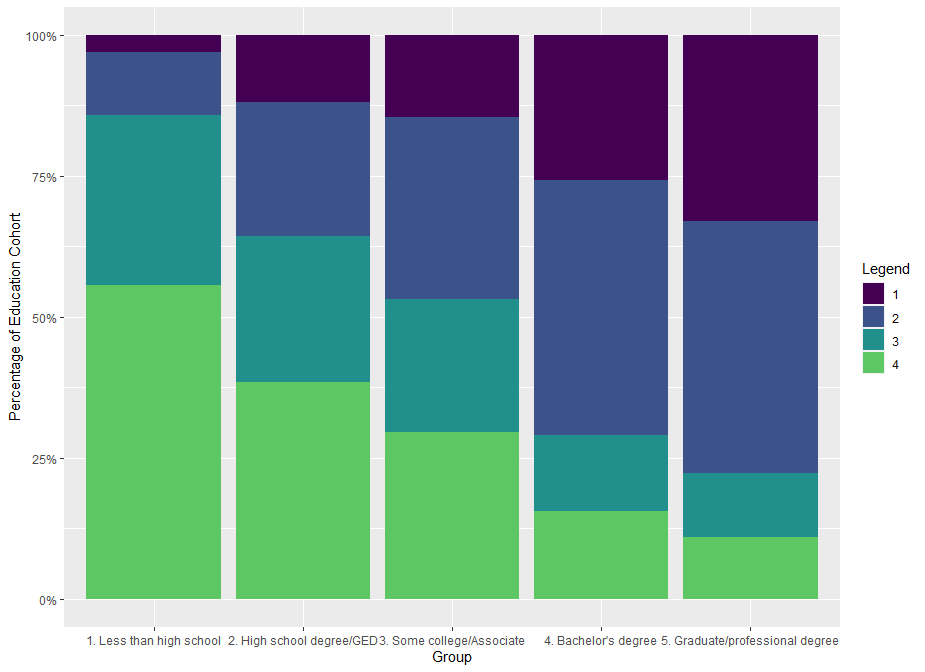


The left bar plot shows the percentage of each group belonging to each education cohort and the right plot shows the percentage of each education cohort belonging to each group.

**S3 Fig 6. Associations between marital status and group.**


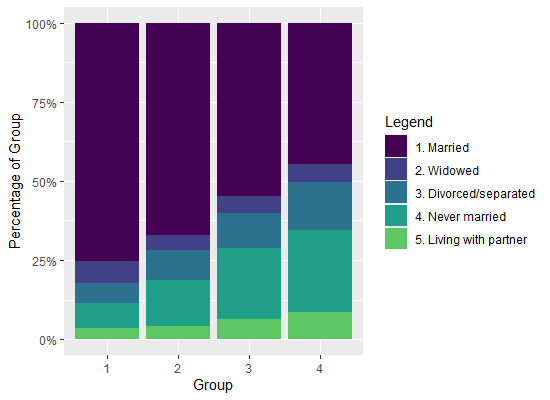

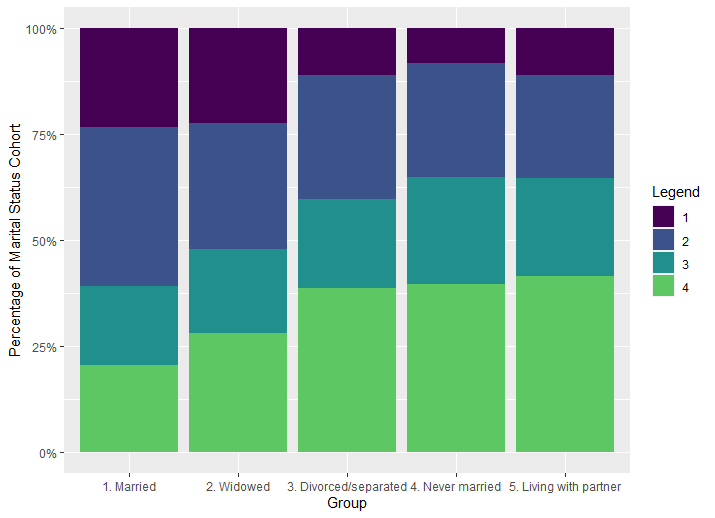


The left bar plot shows the percentage of each group belonging to each marital status cohort and the right plot shows the percentage of each marital status cohort belonging to each group.

**S3 Fig 7. Associations between household income and group.**


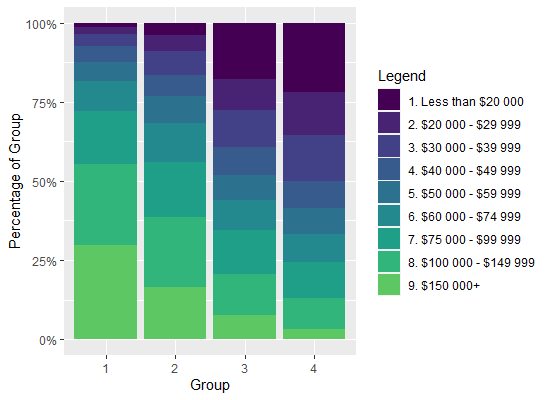

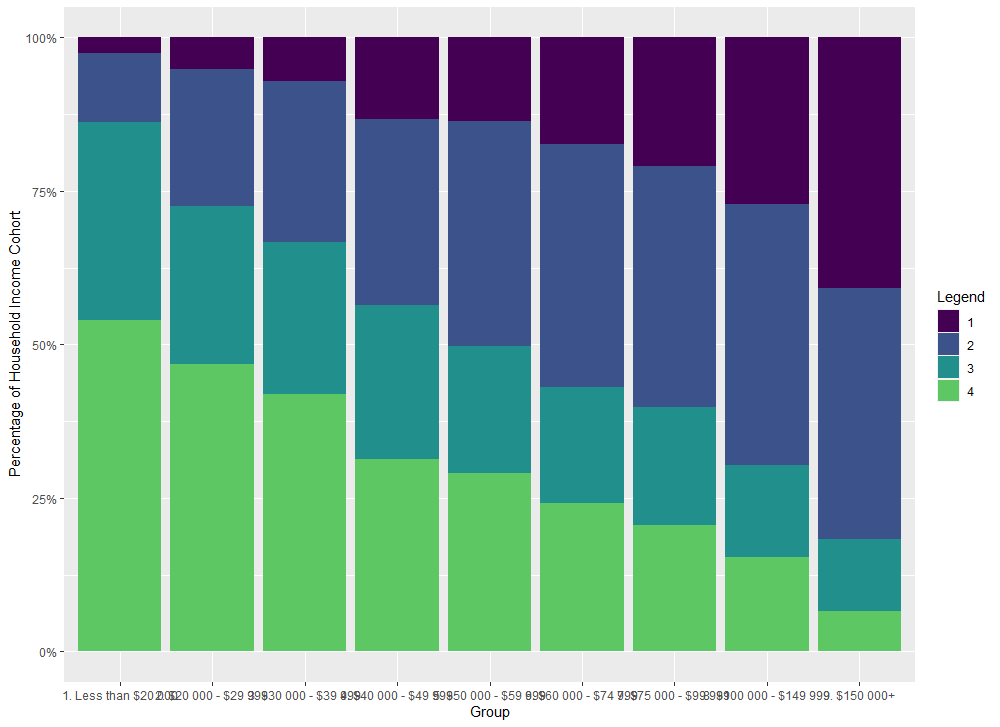


The left bar plot shows the percentage of each group belonging to each household income cohort and the right plot shows the percentage of each household income cohort belonging to each group.

**S3 Fig 8. Associations between financially supporting at least one child and group.**


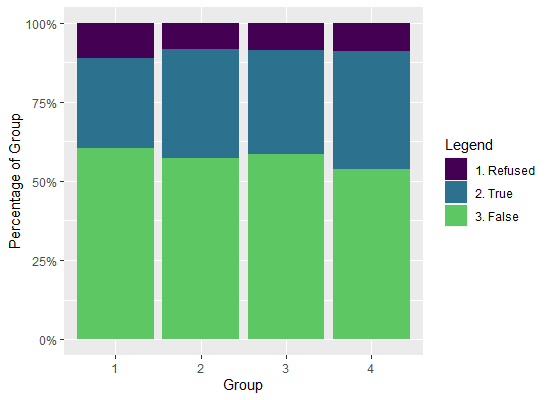

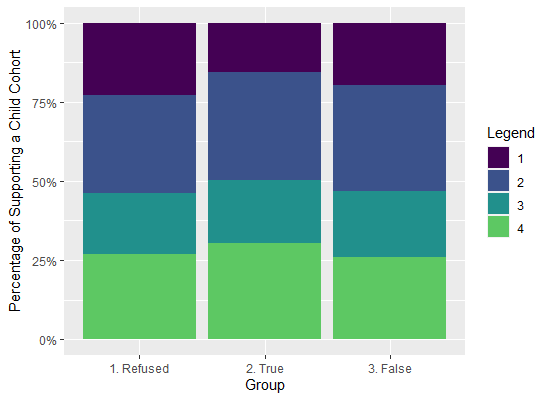


The left bar plot shows the percentage of each group belonging to each supporting a child cohort and the right plot shows the percentage of each supporting a child cohort belonging to each group.
